# Mortality properties of our case series of abdominal aorta aneurysm patients treated with endovascular repair

**DOI:** 10.1186/1749-8090-10-S1-A226

**Published:** 2015-12-16

**Authors:** Serkan Yazman, İsmail Yürekli, Ufuk Yetkin, Levent Yılık, Köksal Dönmez, Hasan İner, Tevfik Güneş, Barçın Özcem, Ali Gürbüz

**Affiliations:** 1 Department of Cardiovascular Surgery, Katip Celebi University Izmir Ataturk Training and Research Hospital, Izmir, Turkey

## Background/Introduction

When we compare EVAR and conventional surgical intervention, EVAR has a better mortality in early period but there was not any significant difference for mortality between EVAR and conventional surgery after 4 years.

## Aims/Objectives

Between 2006 and 2013, 203 abdominal aortic aneurysm patients who were decided to have high risk for conventional surgery and underwent endovascular aortic repair (EVAR) were included in our study.

## Method

Mean age was 69.17 ± 8.83 (between 38-89). Sixteen of these patients were female (7,9%) and 187 of them were male (92,1%). Twelve patients underwent emergency surgery for ruptured abdominal aortic aneurysm.

## Results

Total mortality of 203 patients included in this study after eight years follow-up was 26. Mortality in first month was 14, including eight patients who underwent emergency intervention and with ASA score of IV. Survival rate was 93.2%. Survival rate in 5 years was 84.1% and survival rate of followed-up patients after 8 years was 79.7%. Early period mortality due to aneurysm in patients underwent elective intervention (N: 191) was3.1%. Mortality due to aneurysm in 5 years follow up was 3.6% and survival rate in 8-year follow up was 91.7%.

## Discussion/Conclusion

When compared to conventional surgery, mortality rates are significantly lower in EVAR patients. This is because of less bleeding and lower ischemia period. Debates are ongoing for mortality rates endovascular treatment, but with its minimal invasive nature, it reduces physiological stress and perioperative/postoperative cardiac, pulmonary, and renal mortality is lower. EVAR is the treatment of choice, especially in ruptured abdominal aorta aneurysm.

## Consent

Written informed consent was obtained from the patient for publication of this Case report and any accompanying images. A copy of the written consent is available for review by the Editor-in-Chief of this journal.

